# Tuberculose multifocale chez un immunocompétent

**DOI:** 10.11604/pamj.2015.21.22.6848

**Published:** 2015-05-08

**Authors:** Sanaa Krich, Fatima Zahra Mernissi

**Affiliations:** 1 Service de Dermatologie-Vénérologie, Centre Hospitalier Universitaire Hassan II, Fès, Maroc

**Keywords:** Tuberculose multifocale, immunocompétent, gummy lesions, multifocal tuberculosis, immunocompetent, ésions gommeuses

## Image en medicine

Patient âgé de 34 ans sans antécédents notables qui consulte pour des lésions gommeuses cicatricielles au niveau du visage (A, B) remontant à 2 ans avec une gonalgie droite inflammatoire (C) concomitante et qui présente depuis 9 mois une tuméfaction scrotale gauche douloureuse et fistulisante (D), le tout évoluant dans un contexte de sueurs nocturnes et d'amaigrissement. La biopsie cutanée a objectivé un granulome épithélio-giganto-cellulaire sans nécrose caséeuse. La radiographie standard du genou a montré un pincement fémoro-tibial diffus avec ostéolyse du tibia. L’échographie testiculaire a objectivé une hydrocèle de grande abondance cloisonnée et l’échographie articulaire a objectivé une synovite hypertrophique érodant l'os avec une biopsie synoviale en faveur de tuberculose (TB). Le reste du bilan a objectivé une intradermo-réaction (IDR) positive avec une sérologie VIH négative. Le diagnostic de TB multifocale associant une atteinte cutanée, ostéo-articulaire et génitale chez un immunocompétant a été retenue et le malade a été mis sous antibacillaires sous le schéma 2SRHZ/7RH avec bonne évolution clinique. Les formes multifocales de la tuberculose représentent 9 à 10% des cas et sont l'apanage des patients immunodéprimés principalement porteurs du VIH. Leur pronostic est mauvais avec un taux de mortalité de 16 à 25%. Elles posent en plus un véritable problème de diagnostique, vu son polymorphisme clinique et la diversité des organes pouvant être atteints, ce qui est à l'origine du retard du diagnostic.

**Figure 1:**
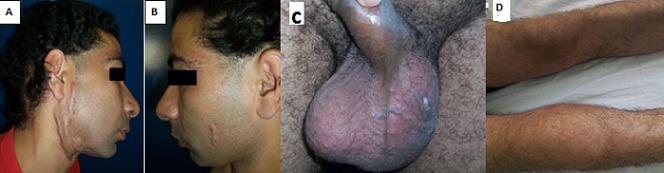
(A,B) gommes en préfistulisation sur le visage; (C) tuméfaction scrotal avec fistulisation et issu de pus; (D) arthrite du genou droit

